# Mid-infrared, super-flat, supercontinuum generation covering the 2–5 μm spectral band using a fluoroindate fibre pumped with picosecond pulses

**DOI:** 10.1038/srep39138

**Published:** 2016-12-15

**Authors:** Maria Michalska, Janusz Mikolajczyk, Jacek Wojtas, Jacek Swiderski

**Affiliations:** 1 Institute of Optoelectronics, Military University of Technology, 2 Kaliskiego Street, 00-908 Warsaw, Poland

## Abstract

Broadband, mid-infrared supercontinuum generation in a step-index fluoroindate fibre is reported. By using ~70-picosecond laser pulses at 2.02 μm, provided by an optical parametric generator, a wide spectrum with a cut-off wavelength at 5.25 μm and a 5-dB bandwidth covering the entire 2–5 μm spectral interval has been demonstrated for the first time. The behaviour of the supercontinuum was investigated by changing the peak power and the wavelength of the pump pulses. This allowed the optimal pumping conditions to be determined for the nonlinear medium that was used. The optical damage threshold for the fluoroindate fibre was experimentally found to be ~200 GW/cm^2^.

Mid-infrared (mid-IR) supercontinuum (SC) sources have recently gained much interest, being of key importance for such applications as molecular fingerprinting^1^, medicine^2^, spectral infrared microscopy^3^ and infrared countermeasures^4^. However, one of the challenges facing this technology is how to obtain broadband light covering a spectral band of at least 3–5 μm, corresponding to an atmospheric transmission window.

Supercontinuum generation is a process in which laser pulses launched into a nonlinear optical medium interact with it, causing the emission of a spectrum that is much broader than the spectrum of the pump pulses. The strength of SC generation depends on the nonlinearity of the medium and the interaction length of light with the medium. Therefore, optical fibres are preferred for continuum generation, providing very good laser beam confinement in a small fibre core area over a long distance.

Silica fibres have been the predominant sources of SC generation to date^5^,^6^,^7^. The longest wavelength that can be generated in this host is below 3 μm because of intrinsic material losses. Therefore, for SC generation beyond this wavelength, fibres with longer infrared transmission windows, along with an appropriate choice of dispersion and nonlinearity are required. To meet these requirements, soft-glass fibres, featuring lower phonon energy than silica fibres, have been adopted as nonlinear media. Mid-infrared SC generation in fluoride^8^,^9^,^10^,^11^,^12^, chalcogenide^13^,^14^,^15^,^16^,^17^ and tellurite^18^,^19^ fibres using different pump schemes has been widely demonstrated recently. Chalcogenide fibres are very interesting media, characterized by high intrinsic nonlinearity and a wide transmission band in the mid-IR, even exceeding 12 μm^20^,^21^,^22^. On the other hand, the material zero dispersion wavelength (ZDW) of this fibre family is located at wavelengths beyond 4.5 μm, which means that direct pumping of the fibres with standard lasers (doped with erbium, thulium, or holmium ions) operating at wavelengths up to 2.1 μm is not optimum for efficient SC generation. The situation is different in case of fluoride fibres, which have already been used for high power, Watt-level, SC generation^8^. Both fluorozirconate (ZBLAN) and fluoroindate (InF_3_) step-index fibres exhibit a ZDW within the wavelength range covered by the most popular and powerful laser systems operating from ~1.5 to 2.1 μm. The advantage of InF_3_ fibres over ZBLANs is a wider transmission band up to 5.5 μm and this feature makes them ideal candidates for SC generation in the 2–5 μm atmospheric window.

Although mid-IR SC generation in fluoroindate fibres is very interesting, only a few demonstrations of such sources have been reported so far. The first report on SC generation in an InF_3_ fibre was in 2013. Theberge *et al*.^23^ used a 9.5-m long 16 μm core fibre (NA = 0.14) with a cut-off wavelength at 2.8 μm pumping it by 70 fs pulses at 3.4 μm. As a result, they achieved the generation of 20 dB spectral flatness SC spanning from 2.7 to 4.7 μm. At the same time our group for the first time demonstrated Watt-level SC generation in an InF_3_ fibre pumped by a 1.55 μm fibre master oscillator power amplifier MOPA^24^ and a 2-μm gain-switched Tm^3+^-doped fibre laser and amplifier^25^. By applying the former pump system, a SC extending from ~1 to 3 μm with 2.09 W of total output average power was achieved. The whole system was tested over the course of a week for several hours a day and no fibre degradation was noticed. In case of the latter configuration, 1.02 W of average power in the band from ~1.7 to 3 μm was obtained. In both cases the long-wavelength edges (~3 μm) achieved were far from the mid-IR transparency limit of fluoroindate glasses (~5.5 μm^26^), which resulted from the pump system performance as well as the parameters of the nonlinear fibre, which had a quite large core diameter (16.7 μm), directly affecting the fibre nonlinear parameter γ and thus efficient spectrum extension. Later, in 2015, Salem *et al*. reported a 1.8-octave wide SC in a dispersion- engineered fluoroindate fibre^27^. By pumping a 7-μm core InF_3_ fibre with a ZDW of 1.9 μm with 100 fs pulses at ~2 μm, they demonstrated a SC spectrum spanning from 1.25 to 4.6 μm. Additional work on SC generation was performed using an InF_3_ fibre pumped by an erbium-doped fluoride fibre amplifier seeded with 400 ps pulses at 2.75 μm^28^. Gauthier *et al*. demonstrated SC generation from 2.4 to 5.4 μm with a maximum average output power of ~10 mW and with up to 82% of the output power corresponding to wavelengths over 3 μm. Despite these impressive results, some issues remain incomplete because they do not answer the question of which wavelength of pump pulses for a particular nonlinear fibre can produce the most efficient SC generation towards the mid-IR. Furthermore, SC spectrum flatness, which is important for many applications, has been also neglected.

In this paper we present experimental results on SC generation in a fluoroindate fibre pumped by 70-ps pulses delivered by an optical parametric generator. Pumping the nonlinear fibre at 2020 nm with a peak power of 111.5 kW injected into the fibre, a SC covering the band of 2–5 μm was demonstrated. Further spectral broadening was limited by the optical damage threshold of the fibre, which was experimentally determined to be ~200 GW/cm^2^. In addition, the influence of the wavelength of pump pulses on SC broadening is also presented. To the best of our knowledge, this is the first demonstration of SC generation in an InF_3_ fibre covering the entire 2–5 μm band while maintaining 5-dB spectral flatness over the whole range.

## Experimental Setup

**Figure 1:**
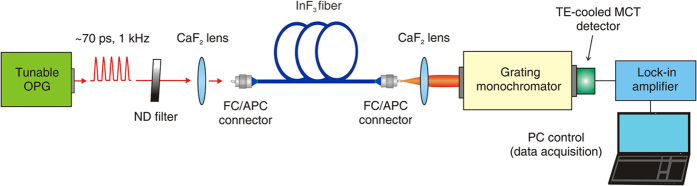
Experimental setup for SC generation in the fluoroindate fibre. OPG-optical parametric generator, ND filter-neutral density filter.

The experimental setup for SC generation in the fluoroindate fibre is shown in Fig. 1.

An optical parametric generator (OPG) consisting of an optical parametric oscillator and amplifier (PG711/DFG-SH, EKSPLA), synchronously pumped by a high power 1064-nm mode-locked Nd:YAG laser (PL2210B-TR-P100 EKSPLA), was used as a pump source. It delivers ~70 ps laser pulses with a nearly Fourier-transform limited linewidth (0.5 cm^−1^) at a repetition frequency of 1 kHz and a maximum pulse energy of 400 μJ at 1.7 μm. The wavelength of the output pulses could be tuned from 1.5 to 16 μm.

**Figure 2:**
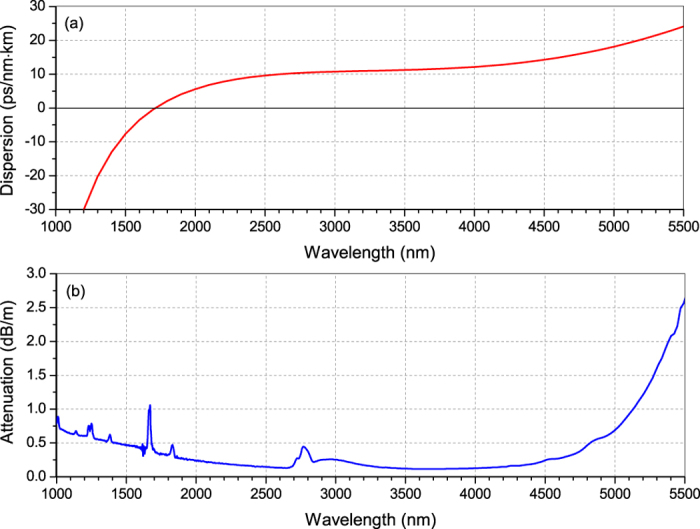
Dispersion (**a**) and attenuation (**b**) curves of the fluoroindate fibre. The data were provided by the manufacturer (Thorlabs Inc).

The step-index, single-mode 9-m long fluoroindate fibre, used as a nonlinear medium, had a core/clad diameter of 9/125 μm, a numerical aperture (NA) of 0.26, and a cut-off wavelength at 3.2 μm. The attenuation and calculated chromatic dispersion curves of the InF_3_ fibre are depicted in Fig. 2. As can be seen, the fibre exhibits losses below 0.5 dB/m for all wavelengths between 1.7 and 4.8 μm with a minimum value (0.115 dB/m) at 3.7 μm. For wavelengths over 4.8 μm, material losses significantly increase, reaching more than 2.6 dB/m at 5.5 μm. The peak near 2.9 μm corresponds to attenuation of the second-order mode. The fibre has a ZDW at 1.72 μm and a flattened dispersion profile (<14 ps·nm^−1^·km^−1^) over the range from 2 to 4.4 μm. Both ends of the nonlinear fibre were cleaved with a diamond stylus and terminated with specialized FC/APC bare fibre adapters, and were finally checked with a microscope to assure good quality of the end facets.

The output of the OPG was coupled to the nonlinear fibre using a CaF_2_ focusing lens, having a focal length of 20 mm. The fluoride fibre output was collimated with an identical CaF_2_ lens. The SC spectrum was measured with 0.2 nm accuracy by a monochromator (iHR-320, Horiba) equipped with a 150-lines/mm diffraction grating, blazed at 2 μm and providing a spectral resolution of 10 nm. A thermo-electrically cooled mercury cadmium telluride (MCT) detector (Vigo System S.A.) with the maximum spectral coverage of 1.8–5.6 μm was employed to measure the spectral fluence. To obtain a high dynamic range for measurements of SC spectra the detector was connected to a lock-in amplifier (SR530, Stanford Research Systems) providing 2% total RMS error. Furthermore, to avoid the effects of high-order diffraction peaks of the grating, appropriate long-pass filters were placed in front of the detection system. The pump pulse energy was measured with an energy meter (LaserStar, Ophir) and a pyroelectric energy sensor (PE10). A continuously variable neutral density filter was used to adjust the pump pulse energy.

## Results and Discussion

**Figure 3:**
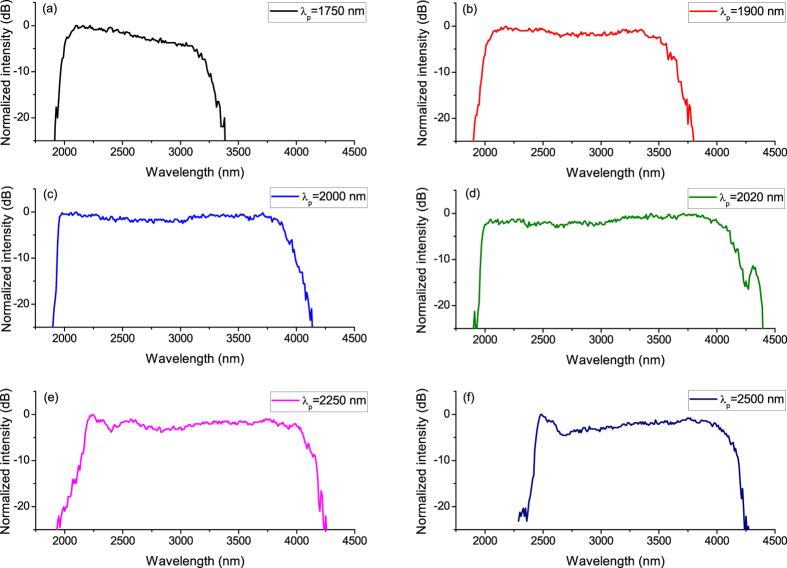
Evolution of the SC spectrum in the InF_3_ fibre for different wavelengths of pump pulses: 1750 nm (**a**), 1900 nm (**b**), 2000 nm (**c**), 2020 nm (**d**), 2250 nm (**e**), and 2500 nm (**f**). The pump pulse energy launched into the fibre was maintained at ~2.7 μJ.

Supercontinuum generation in the InF_3_ fibre was investigated as a function of pump wavelength and pulse energy/peak power launched into the fibre. First, we varied the wavelength in an attempt to maintain the pump pulse energy launched into the fibre at a constant level, set to be ~2.7 μJ (corresponding to ~35 kW of peak power). The aim of this part of the study was to find the optimal pump wavelength for the nonlinear medium that was being used, providing efficient SC spectrum extension towards the mid-IR. The selected pump wavelengths output from the OPG corresponded to the anomalous region of the fiber dispersion, close to the ZDW and also far from this point. Figure 3 plots the evolution of the SC spectra for six different pump wavelengths and for the same energy of pumping pulses injected into the InF_3_ fibre. The recorded spectra were corrected for the detector and grating responsivities. The spectrum of the generated SC was recorded after passing through a long-pass filter with a cut-off edge at 2 μm. The broadest spectrum, spreading to 4380 nm, was measured when pumping at 2.02 μm. Moving the OPG to 2.25 μm and 2.5 μm also provided efficient mid-IR SC generation, but it was narrowed by over 140 nm. Applying shorter wavelengths of 1.75 μm, 1.8 μm, and 2 μm caused the output SC spectra to extend to 3380 nm, 3800 nm, and 4180 nm, respectively. Surprisingly, pumping the nonlinear fibre close to the ZDW (at 1.75 μm and 1.9 μm) did not cause as efficient spectrum extension towards the red wavelengths as in case of the 2.02 μm pump wavelength. It is commonly known that to achieve efficient spectrum broadening towards longer wavelengths, a nonlinear fibre should preferably be pumped in the anomalous dispersion region, relatively close to its zero group velocity dispersion (GVD) point^29^,^30^. However, in our case, the optimum pump wavelength was experimentally determined to be 2020 nm, which is 300 nm away from the calculated ZDW. One possible explanation is that the actual ZDW of the InF_3_ fibre is shifted towards longer wavelengths (close to 2 μm), compared with the ZDW determined from the calculated dispersion curve presented in Fig. 2a. The verification of this issue will be the subject of our further research. Nevertheless, this part of the experiment allowed us to choose the most appropriate pump wavelength for the fluoroindate fibre. The observation of SC evolution at shorter wavelengths (<2 μm) was not carried out, mainly because of detection system limitations.

**Figure 4:**
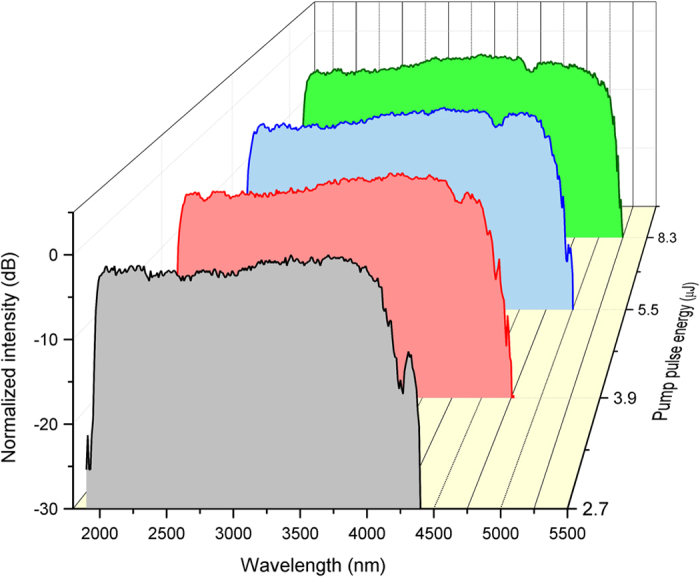
SC spectra obtained from the 9-m-long InF_3_ fibre pumped at 2.02 μm, recorded for injected pump pulse energies of ~2.7 μJ (36.3 kW), 3.9 μJ (52.4 kW), 5.5 μJ (73.9 kW), and 8.3 μJ (111.5 kW).

In the second part of the experiment we examined SC evolution as a function of pump pulse energy, while maintaining the pump wavelength at 2020 nm. Figure 4 shows the resulting SC spectra emitted from the InF_3_ fibre recorded for the pump pulse energies of ~2.7 μJ, 3.9 μJ, 5.5 μJ, and 8.3 μJ with corresponding pulse peak power (assuming a Gaussian shaped pulse) of 36.3 kW, 52.4 kW, 73.9 kW, and 111.5 kW, respectively. The growth of the long-wavelength edge can be observed as a result of increasing the pump energy. For the lowest applied pulse energy, 2.7 μJ, the spectrum extends to ~4.4 μm. The spectral range was considered according to the noise level of the detection system. When the pulse energy was increased to 3.9 μJ, the SC was further broadened by 400 nm into the mid-IR. Applying 5.5 μJ of energy yielded an SC with a spectrum that extended to 5 μm. At the highest coupled pulse energy of 8.3 μJ, the spectrum covered the entire 2–5 μm interval with a cut-off wavelength at 5.25 μm, representing over an octave of optical bandwidth. A signal drop at ~4.2 μm corresponds to absorption by CO_2_ molecules in the detection system. The 5-dB flatness of the spectral intensity, achieved with 8.3 μJ pump pulses, was maintained in the wavelength interval from 2 to 5 μm (a span of 3000 nm), which represents a significant advance compared with other reports on SC generation in fluoroindate fibres^23^,^24^,^25^,^26^,^27^,^28^.

The dynamics of the SC generation in optical fibres is well known. When the fibre is pumped with picosecond or nanosecond pulses in the anomalous dispersion region, modulation instability (interpreted as parametric four-wave mixing) is the main phenomenon in the first step of SC generation. This modulation instability leads to the temporal breakup of pump pulses into a distributed spectrum of many shorter subpulses, which then propagate through the fibre and undergo fission, Raman-induced frequency red-shift, and dispersive wave generation^31^.

As shown in Fig. 4 the spectrum broadens progressively with pump pulse energy until the damage threshold of the fibre is reached, this being the main limitation of further SC broadening. The damage threshold was reached when the energy of the pumping pulses launched into the fibre was ~9.5 μJ. Assuming a Gaussian field profile of the pulse, the damage threshold corresponded to 128 kW of peak power and ~200 GW/cm^2^ of power density on the fibre facet. These levels were additionally verified by pumping three other pieces of InF_3_ fibre, all of which demonstrated similar values for the damage threshold.

## Conclusion

In conclusion, we report, what we believe to be the first demonstration of 5-dB flat SC emitted by a step index fluoroindate (InF_3_) fibre covering the entire 2–5 μm spectral band. Pumping the nonlinear fibre with 70-ps pulses with a peak power of 111.5 kW at 2020 nm yields broadband mid-IR SC generation with a cut-off wavelength at 5.25 μm. The main limitation of further spectrum extension was the damage to the InF_3_ fibre at higher pump pulse energies; the damage threshold was experimentally determined to be ~200 GW/cm^2^. In the near future we are planning to compare the experimental results with numerical simulations. Furthermore, the planned measurements of the dispersion profile of the nonlinear fiber should give us more information on the dynamics of SC generation for different pump wavelengths.

## Additional Information

**How to cite this article:** Michalska, M. *et al*. Mid-infrared, super-flat, supercontinuum generation covering the 2–5 μm spectral band using a fluoroindate fibre pumped with picosecond pulses. *Sci. Rep.* **6**, 39138; doi: 10.1038/srep39138 (2016).

**Publisher's note:** Springer Nature remains neutral with regard to jurisdictional claims in published maps and institutional affiliations.
